# Convulsions in Infants

**Published:** 1833

**Authors:** 


### IV. Convulsions of Young Infants.

We were taught to administer purgatives to new born babes. Our preceptor, a man of extensive obstetrical practice, not only believed in their safety, but in the necessity of their exhibition. His theory was, that the meconium must be purged off with cathartics, or it will irritate the bowels and excite convulsions; which, indeed, were quite common in his practice, and afterwards in our own. Whenever they occurred, we blamed ourselves for not having made a more liberal administration of calomel or castor oil. We have long since discontinued this practice, and seldom see any thing of infantile convulsions in these latter years. It is quite obvious, that they were generally excited by our cathartics, which were not merely superfluous, but positively injurious. It is seldom requisite to give *any* thing to facilitate the expulsion of the meconium, much less an active purgative, the irritation from which, very often excites the spasms it is designed to obviate.

